# Langat Virus Biology and Infection

**DOI:** 10.1002/rmv.70127

**Published:** 2026-03-09

**Authors:** Zinaida Klestova, Daniel Sauter, Michael Schindler, Thomas Iftner

**Affiliations:** ^1^ Institute for Medical Virology and Epidemiology of Viral Diseases University Hospital Tübingen Tübingen Germany

**Keywords:** flavivirus, langat virus, TBEV vaccine, tick‐borne encephalitis

## Abstract

Flaviviruses pose a major threat to global health and can cause severe disease in animals and humans. Among them, tick‐borne encephalitis virus (TBEV) and related members of the tick‐borne encephalitis (TBE) serocomplex are transmitted primarily by ticks and can lead to neurological disease, including encephalitis and death. Despite many years of research on the TBE‐serocomplex, no specific antiviral treatment has been developed. Langat virus (LGTV), another member of this serocomplex typically causes asymptomatic infections in humans, but may cause severe neurological disease in rare cases. Moreover, experimentally LGTV‐infected mice and monkeys show neurological symptoms. While viruses of the TBE‐serocomplex are canonically transmitted by ticks, tick‐independent transmission of LGTV has been reported in mice, suggesting a broader transmission potential. Notably, the full host range, vector competence and global distribution of LGTV remain poorly defined, and climate change‐driven expansion of tick vectors may enable the emergence of this virus in new regions. In this review, we summarise our current knowledge of LGTV, focussing on vector competence, transmission routes, tissue and host tropism, pathogenicity and animal models. Furthermore, we discuss the potential application of LGTV as a vaccine candidate for TBEV. By highlighting the unique features of LGTV and identifying current research gaps, we aim to inspire interest in this understudied virus and its value as a tool to uncover fundamental aspects of tick‐borne flavivirus biology and strategies for vaccine development.

## 1 Introduction

Flaviviruses are significant pathogens, with some of them causing severe disease in animals and humans. Among them, TBEV and related members of the TBE‐serocomplex are transmitted primarily by ticks. They are found across large areas of the Eurasian continent, causing several thousand cases of TBE in Europe every year [1, 2, 3]. Symptomatic cases include a range of neurological manifestations, including meningitis, encephalitis, paralysis or even death [4, 5, 6, 7]. Estimates for mortality rates of TBEV range up to 35%, depending on the subtype [8, 9]. Notably, viruses of the TBE serocomplex also benefit from climate change and continue to emerge in new geographic regions. This includes TBEV, whose prevalence has increased over the past 30 years [9].

The TBE‐serocomplex is a group within the genus *Orthoflavivirus* (renamed from *Flavivirus* in 2022) [10] and comprises several viruses of clinical relevance, including different subtypes of TBEV (i.e., European, Far Eastern, Siberian, Obskaya, Himalayan, Baikalian‐1 and Baikalian‐2) [6, 11, 12], Omsk haemorrhagic fever virus (OHFV), Powassan virus (POWV), Kyasanur Forest disease virus (KFDV) and the closely related Alkhurma haemorrhagic fever virus (AHFV or ALKV) and Nanjianyin virus, as well as Louping ill virus (LIV), and Langat virus (LGTV) (Table 1) [14, 15].

**TABLE 1:** Members of the TBE‐serocomplex.

Virus	Acronym	Official ICTV species name [13]
Tick‐borne encephalitis virus	TBEV	*Orthoflavivirus encephalitidis*
Kyasanur forest disease virus	KFDV	*Orthoflavivirus kyasanurense*
Alkhurma (haemorrhagic fever) virus	AHFV or ALKV	
Langat virus	LGTV	*Orthoflavivirus langatense*
Louping ill virus	LIV	*Orthoflavivirus loupingi*
Omsk haemorrhagic fever virus	OHFV	*Orthoflavivirus omskense*
Powassan virus	POWV	*Orthoflavivirus powassanense*

LGTV represents a particularly intriguing but underexplored member. It is closely related to OHFV and KFDV [16] and shares 82%–88% amino acid identity with TBEV [17, 18]. Notably, LGTV and other TBE‐serocomplex viruses share common envelope glycoprotein epitopes, which often leads to cross‐reactive immune responses [4, 17, 18, 19, 20, 21]. These observations have also led to the experimental use of LGTV as a preventive vaccine against TBEV [22].

LGTV is generally considered lowly pathogenic, causing mainly asymptomatic and mild infections [15, 23]. However, experimental and clinical observations indicate that it can infect neurons and astrocytes [24, 25, 26, 27], thereby affecting central nervous system (CNS) function. In animals, LGTV infection has been associated with memory deficits, altered anxiety behaviour, and neurological disease resembling TBEV infection [26, 28, 29]. Furthermore, a biphasic course of disease has been observed in a small number of LGTV‐infected humans, similar to the European and Siberian subtypes of TBEV [19, 22, 30]. An initial phase of flu‐like symptoms is followed by a second phase of headache, myalgia and neurological dysfunction, including rare cases of encephalitis [19, 22, 30, 31].

Given its ability to infect the CNS, cross‐reactivity with other flaviviruses, and potential as a vaccine model, LGTV warrants further study. We summarise our current knowledge on LGTV, focussing on its tropism, transmission routes, pathogenesis, and relevance as a model for TBEV research.

## 2 Natural Hosts, Vectors and Transmission Routes of LGTV

LGTV was discovered in Malaysian ticks (Ixodes granulatus) and forest rats (*Rattus sabanus, Rattus rajah, and Rattus muelleri*) by Smith et al. near Kuala Lumpur in 1956 (Table 1) [32]. The first wild‐type strain of LGTV was termed TP21 [32]. A high prevalence of antibodies against LGTV has subsequently been observed in several rat species (*Rattus bowersi, Rattus sabanus, Rattus muelleri, Rattus rajah*), suggesting that they are involved in the natural cycle of transmission [15]. The virus is not known to cause any disease in the above‐mentioned forest rats [14]. However, studies determining the tropism and pathogenicity of LGTV in rodents and other mammals are limited.

**FIGURE 1:**
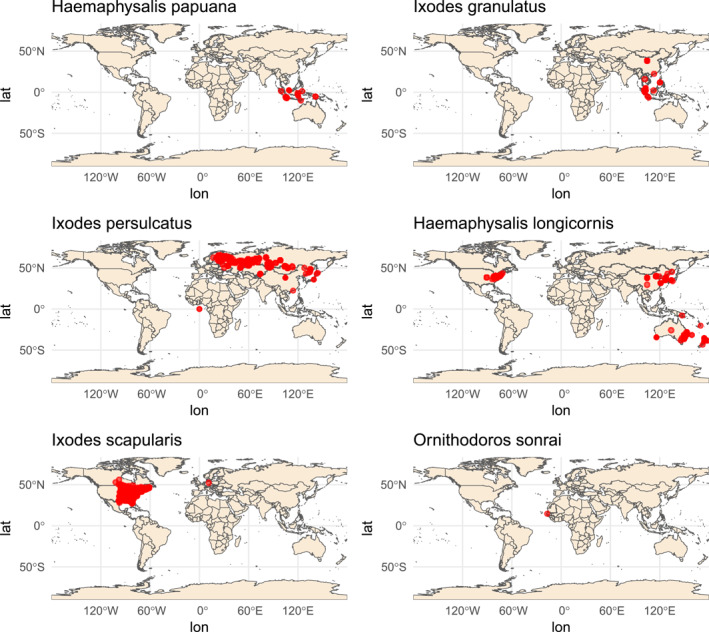
Global distribution of tick species that are experimental or natural vectors of LGTV. Distributions are based on occurrence records retrieved from the Global Biodiversity Information Facility (GBIF.org (29 August 2025)GBIF Occurrence Download: https://doi.org/10.15468/dl.xpjnvh. Accessed from R via rgbif (https://github.com/ropensci/rgbif)). Red dots indicate reported collection sites for *Haemaphysalis papuana*, *Ixodes granulatus*, *Ixodes persulcatus* (known natural vectors), as well as *Haemaphysalis longicornis*, *Ixodes scapularis*, and *Ornithodoros sonrai* (experimental vectors) Maps were generated in R using the packages *rgbif*, *ggplot2*, and *rnaturalearth*. Please note that the maps illustrate the geographical range of selected available records and may not fully reflect the true distribution of each species due to sampling and reporting biases.

In the following years, LGTV has been isolated from additional tick species, including *Haemaphysalis papuana* (Thailand) [38] and *Ixodes persulcatus* (Central Siberia, Russia) [15] (Table 2). Furthermore, experimental infection showed that *Haemaphysalis longicornis* [33, 34], *Ixodes scapularis* [35], and *Ornithodoros sonrai* [36, 37] are also susceptible to LGTV infection. These tick species are found around the globe (Figure 1).

**TABLE 2:** LGTV vectors.

	Scientific name	Common name	Geographic distribution
Natural vectors	*Haemaphysalis papuana* [15]		South Asia, South‐East Asia, Australia
	*Ixodes granulatus* [32]	Asian rodent tick	East Asia, South‐East Asia
	*Ixodes persulcatus* [15]	Taiga tick	North‐East Europe, central Asia, East Asia
Experimental vectors	*Haemaphysalis longicornis* [33, 34]	Asian longhorned tick, longhorned tick, bush tick, Asian tick, or cattle tick	East Asia, central Asia, Australia, New Zealand, Eastern USA
	*Ixodes scapularis* [35]	Black‐legged tick, bear tick, deer tick	Eastern half of North America
	*Ornithodoros sonrai* [36, 37]		West Africa

**FIGURE 2:**
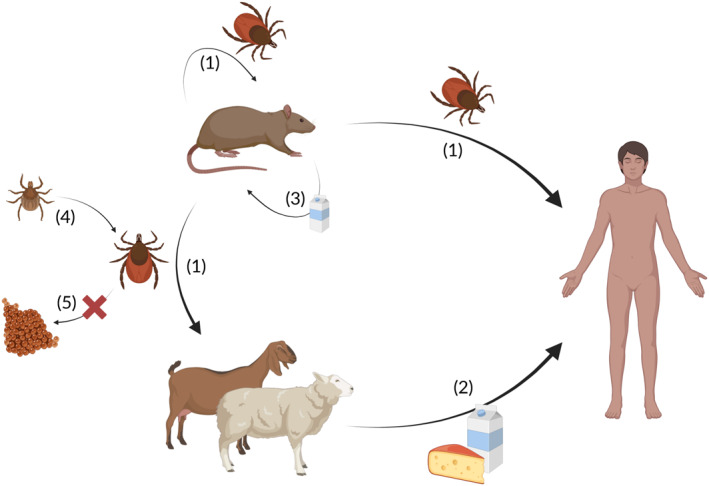
Transmission routes of LGTV. (1) Ticks transmit the virus between rodents, humans and most likely other mammalian hosts such as sheep or goats. (2) Dairy products such as raw milk may potentially serve as tick‐independent transmission vectors to humans. (3) Experiments in mice also demonstrated tick‐independent transmission via the gastro‐intestinal route. (4) LGTV can persist during different stages of tick development, but is most likely not transmitted vertically from adult ticks to their eggs (Image generated with biorender).

In line with ticks being the main vectors, efficient transmission of LGTV has been demonstrated from experimentally infected mice to *Haemaphysalis longicornis* ticks (Figure 2) [34]. Intriguingly, mice can also serve as a ‘transmission bridge’, enabling the transmission of LGTV from infected ticks to adjacent naïve ticks, co‐feeding on the same mouse [34]. Maximum titres in adult *H. longicornis* ticks infected with the TP21 strain were observed 28 days after virus inoculation [33]. Notably, the life span of ticks can be several months or even years [39], and *Ornithodoros sonrai* ticks experimentally infected with LGTV by feeding on infected mice have been shown to be capable of virus transmission after more than 3 years [36, 37] In agreement with the long‐term persistence of LGTV in *Ornithodoros sonrai,* transstadial transmission of LGTV has been demonstrated from experimentally infected larvae to adult *Ixodes scapularis* ticks [35], which allows the virus to persist in the tick's body over different developmental stages, despite molting of its host. In contrast, one study failed to demonstrate vertical LGTV transmission from infected adult ticks to their progeny (i.e., transovarial transmission) [34].

Humans are incidental hosts of LGTV and other members of the TBE‐serocomplex, becoming infected through tick bites or (potentially) consumption of unpasteurised milk and milk products from infected mammals (Figure 2) [14, 30, 40, 41, 42, 43, 44]. While there are no direct reports of LGTV transmission through milk to humans, vertical transmission of the virus through rodent milk has been demonstrated [45]. In mice, LGTV can be transmitted to offspring via maternal milk, and LGTV infection causes tissue lesions in the mammary gland where viral particles are present [45]. While pasteurisation fully inactivates the virus, LGTV remains infectious in raw goat milk at refrigeration temperatures and after a cheese‐making‐like thermal regimen [40]. This underscores the plausibility of alimentary transmission scenarios of LGTV. Contact‐dependent transmission in the absence of ticks was observed from infected to uninfected sentinel IFNAR1‐deficient mice, high levels of LGTV RNA have been found in saliva, stool, faeces and urine [28]. These findings demonstrate that LGTV cannot only be transmitted by a variety of tick genera, but also independently of the arthropod vector.

## 3 Cell and Tissue Tropism of LGTV

LGTV replicates in several cells and tissues of different tick and mammal species. The main site of LGTV replication in *H. longicornis* ticks is the midgut cells, but replication in other tissues, including salivary glands and neural tissues (synganglion) [33, 34].

Following peripheral inoculation of wild type mice, LGTV can be detected in cells of the blood, skin and brain, including neurons and astrocytes [35]. LGTV does not replicate in human peripheral leucocytes [46]. In contrast, TBEV replicated efficiently in peripheral leucocytes from a recovered TBE patient and in cells from an individual without pre‐existing immunity [46]. Table 3 provides a comprehensive overview of cell lines and primary cells that are susceptible and/or permissive to LGTV infection.

**TABLE 3:** Cells that are susceptible to LGTV strains and derivatives thereof.

	Species	Cell type/cell line
Arthropod cells	*Ixodes scapularis*	IDE8 (embryonic cell line) [47]
	*Ixodes ricinus*	IRE/CTVM19 (embryonic cell line) [47]
	*I. scapularis*	ISE6 (embryonic cell line) [48, 49, 50]
	*Haemaphysalis longicornis*	Midgut cells [33]
	*H. longicornis, I. scapularis*	Salivary glands [34, 35]
	*H. longicornis*	Synganglion cells [34]
Mammalian and avian cells	Mouse (*Mus musculus*)	Astrocytes [25, 26, 27, 51]
		Cells from lymphoid organs (lymph nodes, thymus) [51]
		Cerebral cortex cells [27, 51]
		Choroid plexus epithelial cells [27]
		Dendritic cells [25, 51]
		Endothelial cells [27]
		Liver, lung, kidney, thymus, lymph nodes, spinal cord, brain cells [27, 51, 52, 53]
		Macrophages [51]
		Mammary gland epithelial cells [25, 26, 45, 51]
		Microglia [25, 26, 27]
		Neurons (hippocampal) [26, 27]
		Neuro‐2A (neuroblastoma cells) [54]
		Olfactory bulb cells [25]
		Oligodendrocytes, neutrophils, monocytes, stromal cells, Medulla oblongata cells [25, 51]
		Pericytes [27]
		Salivary gland cells [51]
		Spleen cells (splenocytes) [50, 51, 55]
		Vascular leptomeningeal cells [27]
	Wistar rats (*Rattus norvegicus*)	Astrocytes [29]
		Microglial cells [29]
	Chicken (*Gallus gallus*)	Primary embryo cells [54, 56, 57]
	Rhesus macaque (*Macaca mulatta)*	LLC‐MK‐2 (kidney epithelial cells) [58]
	African green monkey (*Chlorocebus* sp.)	Vero cells (kidney epithelial cells) [56, 57, 59, 60, 61, 62, 63]
	Hamster, syrian golden *(Mesocricetus auratus)*	BHK‐21 (baby hamster kidney) [64]
	Human (*Homo sapiens*)	Astrocytes [24, 26]
		HEK293T cells [49]
		HeLa (cervical epithelial cells) [65, 66]
		NCI60 cells [67]
		Neurons [24]
		SHSY5Y (neuroblastoma cells) [54]
		WI26 (lung cells) [65]

## 4 Persistent LGTV Infection in Cell Lines

Intriguingly, the cytopathogenicity of LGTV differs between tick and mammalian cells. In an *Ixodes scapularis* embryonic cell line (ISE6 cells) LGTV infection persists without strong CPE [49, 50]. This is in contrast to HEK293T (human embryonic kidney) cells, where massive cell death occurs within days of infection [49, 68, 69]. However, a small fraction of infected HEK293T cells survives and remains persistently infected for months [49, 68, 69]. The persistently infected cultures do not undergo lysis and are morphologically similar to uninfected cells. Still, ultrastructural changes associated with infection, including ER membrane rearrangements and convoluted membranes are observed [69]. Furthermore, differential transcriptome profiles of acute phase compared to persistently infected LGTV HEK293T cells revealed virus‐induced perturbation of apoptosis, which may be critical for persistent infection [68].

Experiments in tick cells revealed no changes in viral RNA sequence during acute infection or at the onset of persistence. However, defective viral genomes were detected after five weeks of serial cell passage in Vero cells [49]. Two synonymous nucleotide changes were identified in the *E* coding region at this time point [49]. In HEK293T cells, synonymous nucleotide changes were detected in the region encoding NS2B during LGTV persistence (at 70 dpi) [69]. The significance of these nucleotide changes for viral persistence have remained unclear. At late, but not early stages of persistent infection of HEK293T cells, virus production is also accompanied by the production of defective interfering particles (DIPs) [69]. Although DIPs are a feature of persistent infection in cell culture, they are not required to initiate persistent infection [69].

These observations highlight that LGTV can persist in both arthropod and mammalian cells, albeit with distinct cellular outcomes. While persistence in cell culture is well documented, the mechanisms and relevance of LGTV persistence in vivo, in either ticks or mammalian hosts, remain to be determined.

## 5 Host Cell Factors and Environmental Factors Determining LGTV Replication

Host cell proteins, glycans or glycolipids may serve as cellular attachment and entry receptors, and it has been suggested that LGTV binds to different receptors in different cell types [67]. Binding of LGTV to a panel of 60 tumour cell lines (NCI60 cell panel) was variable, even between cells from the same cell type or organ [67]. Notably, relatively low viral binding to CNS cell lines was observed compared to control A549 cells. The highest binding was observed for the SW620 colon cancer cell line [67]. In contrast to many other flaviviruses, for which heparan sulphate (HS) is the major attachment determinant, LGTV binding to A549 cells is not dependent on the presence of HS, gangliosides, or sialylated glycans, but instead on specific proteins or a glycan component present on these proteins [67]. Structural studies of the LGTV *E* protein further suggest that conformational flexibility in the receptor‐binding domain may contribute to host cell entry [70]. More specifically, two different crystal morphologies of the LGTV *E* glycoprotein have been detected [70].

Apart from cellular attachment and entry factor interactions, host immune responses strongly influence LGTV susceptibility and tropism. For example, *IFNAR1* knockout mice revealed that interferon responses restrict LGTV replication [51]. In line with a strong selection pressure exerted by IFN responses, LGTV suppresses the gp130/JAK/STAT signalling pathway [71]. Vice versa, tick‐derived molecules such as Peroxiredoxins are required for efficient replication in host cells (BHK), and increase LGTV titres by an unknown mechanism [64]. Heat shock proteins (HSP70, HSP90, gp96), complement associated protein factor H and trypsin have also been implicated in supporting LGTV replication in different cell lines from *Ixodes scapularis* (IDE8) and *Ixodes ricinus* (IRE/CTVM19), suggesting that cellular stress responses are exploited during infection [47]. LGTV infection also triggers the unfolded protein response (UPR) and alters autophagic flux [72]. Notably, LGTV is sensitive to PKR‐like endoplasmic reticulum kinase (PERK), an ER protein that regulates the unfolded protein response, suggesting that PERK is a potential mediator of the intrinsic immune response to LGTV [72]. For TBEV, glutathione S‐transferase (GST) molecules have been suggested to play an important role in viral replication [48]. The authors suggested that ubiquitous GSTs are used by cells and the virus for mutual survival and proliferation. In the case of LGTV, increased cell death (ISE6 tick cells) was detected when the *Ixodes scapularis GST1* gene was knocked down, indicating a similar role for GSTs in TBEV and LGTV infection [48].

Besides host cell factors, LGTV tropism and replication are also be determined by environmental factors such as temperature. Rumyantsev and colleagues generated variants of LGTV using 5‐fluorouracil and tested their temperature sensitivity and tropism, with the ultimate goal of isolating a strain that lacks neuroinvasiveness/neurovirulence [54]. They identified one strain (E5‐104), whose growth in neuroblastoma cells was restricted at 32°C (vs. 37°C), but still replicated efficiently in Vero cells at this temperature [54].

Thus, successful LGTV replication is determined by a range of host receptors, innate immune responses, cellular stress pathways and environmental factors such as temperature.

## 6 Pathogenesis and Animal Models

After severe acute TBEV infection patients may suffer from debilitating neurological sequelae, which can be the result of viral persistence and infection‐induced neuronal damage, [6] although the underlying pathomechanisms have remained incompletely understood. Even less is known about the pathogenicity and pathomechansisms of LGTV.

LGTV effectively infects wild type mice. In an early study, intracerebral inoculation of Swiss A2G mice with LGTV strain TP21 resulted in 100% mortality in both adult and suckling mice. Upon intraperitoneal infection, about 90% of adult mice survived, whereas baby mice all died [52]. LGTV neuroinvasion likely occurs by direct infection of microvascular endothelial cells [63]. In C57BL/6 mice, higher LGTV replication (strain TP21) and increased lethality were observed upon suppression of type I IFN responses [51]. LGTV was detected in peripheral organs, spinal cord and brain when the interferon pathway was blocked, but not in wild‐type mice brains [51]. Notably, however, neuroinvasiveness needs to be clearly distinguished from neurovirulence, and the presence of tick‐borne flaviviruses in the brain does not necessarily result in symptomatic disease.

Impaired learning and memory formation, as well as altered anxiety behaviour have also been observed in wild type C57BL/6 J (WT) mice infected with LGTV TP21 [26]. These neurological symptoms were associated with changes in hippocampal neuron morphology, a significant decrease in neuronal spine density, as well as microglial activation and astrogliosis [26].

Histological analyses also revealed virus‐induced rearrangements of subcellular compartments: [52] the nucleoplasm became denser and the cytoplasm of infected cells became granular as infection progressed. Within the cytoplasm, dense bands formed because the membranes of adjacent vacuoles were often squeezed together as the ER and Golgi apparatus were greatly swollen [52]. LGTV particles were found in small irregularly shaped vesicles located within the cisterns of ER. The developing and mature virus was detected only in the glial cytoplasm and in the extracellular spaces associated with these cells [52].

Of note, co‐infection experiments with unrelated togaviruses (Semliki Forest virus or Sindbis virus) reduced LGTV lethality in mice when administered before LGTV challenge, suggesting possible interference between arboviruses during sequential infection [73, 74].

Apart from mice, rats also show symptoms after LGTV infection. More specifically, LGTV infection of Wistar rat pups leads to gait disturbance, hypokinesia and weight loss. [29] The brains of animals with LGTV encephalitis showed signs of inflammation in the thalamus, hippocampus, midbrain, frontal pole and cerebellum [29]. Furthermore, levels of interferon‐β and ‐γ, interleukin‐6, monocyte chemotactic protein‐1 and RANTES were increased in the cerebrospinal fluid of infected animals [29].

Comparative analyses of TBEV an LGTV revealed that both viruses can cross human endothelial monolayers transcellularly without overt barrier disruption [75]. TBEV crosses the barrier more efficiently in this model suggesting quantitative rather than categorical differences [75]. Furthermore, TBEV outcompetes LGTV and achieves higher replication in neuronal/glial cultures [76]. Within the TBEV clade, the *E* protein is a key determinant of neuronal entry and neurovirulence in vivo, as demonstrated by experiments using chimaeric viruses with swapped *E* genes [77, 78]. However, replacing LGTV prM plus the *E* ectodomain with those of TBEV results in a lowly pathogenic chimera with LGTV‐like cortical tropism, indicating that prM/E is insufficient to confer TBEV‐like neurovirulence to LGTV [79].

Notably, neuron‐ and astrocyte‐intrinsic IFN responses (including viperin) efficiently suppress LGTV replication and spread within the CNS [80, 81, 82]. Comparative transcriptomic profiling suggests that LGTV induces a weaker host response than TBEV, particularly in neurons and astrocytes, which is in line with less efficient replication [27]. While LGTV triggers interferon signalling and inflammatory pathways, the magnitude of dysregulation is markedly lower than that caused by TBEV, which may explain its reduced pathogenicity. Cytotoxic T cell responses have also been implicated in the pathogenicity of LGTV, as LGTV‐sensitised lymphocytes can damage non‐neuronal brain cells in vitro [83].

In summary, these findings demonstrate that LGTV can cause neuropathological changes and symptoms in susceptible animal hosts, with disease severity strongly influenced by the route of infection and intact IFN responses. While LGTV is clearly less pathogenic than TBEV, it provides a valuable model to investigate pathogenesis and host–virus interactions in tick‐borne flavivirus infections. Current data support a model in which TBEV's superior neuronal replication, together with more effective evasion of CNS‐intrinsic IFN responses results in higher neurovirulence and broader CNS distribution compared to LGTV.

## 7 Natural Isolates and Attenuated Strains of LGTV

LGTV is a lipid‐enveloped virus that is not perfectly spherical in shape with an inner icosahedral protein shell [52]. LGTV particles are about 40–68 nm in diameter [52]. The viral genome consists of approximately 11 kb positive‐sense, single‐stranded RNA [59, 84]. As a prototypical flavivirus, the LGTV RNA genome encodes a polyprotein that is proteolytically processed into the mature viral proteins [85, 86]. These include the structural proteins envelope (E), capsid (C), and precursor membrane (preM), as well as the non‐structural proteins NS1, NS2A, NS2B, NS3, NS4A, NS4B and NS5. These proteins are required for virion assembly genome replication and immune evasion. Among them, the *E* protein is an important virulence factor in the TBE‐serocomplex [87]. Mutations in the *E* protein have been implicated in attenuating neuroinvasiveness, although no single mutation has yet been identified that specifically affects the ability of the virus to infect neurons [87].

Different LGTV strains vary considerably in their characteristics, particularly in the degree of neurovirulence. Nevertheless, TP21 is the only natural isolate that has been extensively characterised in a wide range of possible hosts and vectors [73, 88, 89]. Virtually all known laboratory strains are derived from this isolate, including TP21_I, TP21_II, TP21_III, and Elantcev15‐20/3 [22, 30, 57, 58, 61, 66, 90].

Reverse genetics has further advanced LGTV research. Initially, it has not been possible to produce full‐length infectious TP21 cDNA from cDNA fragments cloned in *E. coli*, most likely due to inactivating deviations from the consensus sequence [66]. Later efforts succeeded in rescuing the so‐called E5 strain from cDNA, and rescued viruses harboured minimal changes compared to the consensus sequence [66].

These studies underscore the importance of both natural genetic variation and engineered cDNA‐derived clones in LGTV research. The TP21 isolate and its derivatives provide insight into attenuation and host tropism, while reverse genetics represents a valuable tool for vaccine development and targeted mutagenesis.

## 8 Potential Use of LGTV as TBEV Vaccine

As LGTV is considered largely non‐pathogenic in humans, the development of a LGTV‐based live vaccine against TBEV was studied in the former Soviet Union in the 1970s (Table 4) [22, 56]. About 650,000 doses of the TP21‐derivative Elantcev 15‐20/3 were administered [19, 22]. While initially promising, several recipients developed biphasic courses of disease with neurological complications following vaccination, ultimately resulting in the withdrawal of the vaccine [19, 22, 30]. More specifically, 32 cases of meningoencephalitis with permanent neurological sequelae developed among 379,540 LGTV vaccinees that had no previous TBEV vaccination and three cases in a group of 269.939 individuals that had previously received an inactivated TBEV vaccine [22]. Clinically, the cases of vaccine‐induced encephalitis resembled those caused by TBEV (European strain) [22, 30]. Notably, Elantcev 15‐20/3 also caused neurological lesions in 30% of monkeys upon intracerebral inoculation of the virus [22, 30].

**TABLE 4:** LGTV‐derived vaccine candidates against TBEV.

Vaccine candidate	Origin	Virulence	Protection, outcome	Outcome
Elantcev 15‐20/3 [22, 30]	TP21 derivative; used in 1970s in USSR (∼650,000 doses)	Caused biphasic disease and meningoencephalomyelitis in some vaccinees	Induced immunity against TBEV	Withdrawn due to safety concerns
E5 [91]	TP21 attenuated by passaging in chicken embryos + Vero cells	∼2000 times less neuroinvasive than TP21; low neurovirulence in monkeys; genetically stable	Long‐lasting protection in mice (18–24 months)	Prototype for attenuated vaccine
E5‐104 [45]	Subclone of E5	∼10^3^‐fold reduced replication versus TP21; not neuroinvasive in immunodeficient mice		
E5‐651 [70]	Subclone of E5	∼340× less virulent and ∼5100× less neuroinvasive than TP21		
T/1674‐mirV2 [92]	LGTV backbone + prM/E from TBEV Sofjin strain	Apathogenic in several mouse models	Single dose protected mice; antibody titres comparable to licenced vaccines	Failed to balance safety and efficacy
LGT/DEN4 [55, 56, 93, 94]	Chimaeric LGTV (TP21) expressing structural proteins of DENV type 4	5000 times less neurovirulent than the parental LGTV strain in mice; pathogenic in monkeys	Induces protection against LGTV strain TP21 in mice, TBEV protection unknown	Pending

Subsequent vaccine studies focused on the strongly attenuated LGTV strain E5. It was generated from TP21 via repeated passaging (42 times) in chicken embryos and an additional passage in monkey Vero cells [57, 60, 61]. E5 is 2000 times less neuroinvasive than the parental TP21 strain in immunocompetent mice [61]. Furthermore, it is characterised by low neurovirulence in *Ateles geoffroyi* monkeys inoculated intracerebrally and in *Macaca mulatta* monkeys inoculated intraspinally [61]. The complete genome sequences of E5 and the parental TP21 strain have been determined, revealing nucleotide differences that result in amino acid substitutions in *E*, NS3 and NS5 [54, 56, 66]. Overall, the LGTV E5 strain has six mutations that distinguish it from the virulent parental virus. E5 remains stable during passaging in animals and in cell culture [95]. Immunisation with the E5 strain protects mice against the Far Eastern TBEV subtype for 18–24 months upon challenge with lethal viral doses [91]. E5‐derivatives such as E5‐104 and E5‐651 show even further reduced replication efficiency and neurovirulence (even in immunodeficient mice), [54, 61] providing a useful basis for further development of a live attenuated vaccine against TBEV.

To further avoid vaccine induced‐adverse effects while maintaining immunogenicity, efforts were made to develop a vaccine based on a chimaeric LGTV. One candidate (called T/1674‐mirV2) replaced the LGTV prM/E genes (strain T‐1674) by the respective orthologs from the wild‐type Far Eastern subtype TBEV strain Sofjin [92]. As additional safety precaution, neuropathogenicity of this virus was suppressed using CNS‐enriched microRNAs that target the viral genome [92]. The chimaeric vaccine strain was immunogenic in mice and protected them from lethal TBEV infection [92]. Antibody levels produced after a single dose were similar to those produced by the administration of three doses of the two inactivated TBEV vaccines currently approved in Europe [92].

Another chimera (LGT/DEN4), combining LGTV with Dengue virus type 4 structural proteins resulted in a 5000‐fold reduction in neurovirulence compared to the parental LGTV in suckling mice and caused a 714,000‐fold reduction in neuroinvasiveness [55, 56, 93, 94]. However, it still caused neurological symptoms in one of four African Green monkeys upon intracerebral infection [93]. While this chimaeric virus protected mice from fatal LGTV infection, its effects on TBEV infection remain to be determined [56].

Intriguingly, LGTV was also briefly used in the 1960s as an oncolytic agent to treat cancer patients. However, two out of 28 patients receiving LGTV (24 x strain TP‐21, 4 x strain TP‐77) developed fatal encephalitis [31], highlighting the LGTV pathogenic potential in vulnerable risk groups (such as leukemia patients).

Today, safe and effective TBE vaccines in Europe and Russia rely on formalin‐inactivated whole virus preparations produced in cell culture. Nevertheless, the discovery of new and divergent TBEV subtypes (such as Obskaya, Himalayan, and Baikalian lineages) highlights the continuing need for improved vaccine strategies. Live‐attenuated LGTV‐derived or chimaeric viruses remain conceptually attractive because of their ability to elicit strong and durable immunity, but safety concerns have thus far prevented clinical translation. Notably, experiments in mice revealed that LGTV vaccination protects from TBEV‐induced disease, but does not prevent TBEV entry into the brain [89, 96].

In summary, the history of LGTV as a vaccine candidate is a striking example of the fine line between attenuation and pathogenicity. The lessons from LGTV caution that any approach in vaccine design must carefully balance safety with protective efficacy.

## 9 Conclusion

Despite its discovery more than half a century ago, the biology, epidemiology, and pathogenic potential of LGTV remain incompletely understood. Its natural distribution and circulation in reservoir and vector species remain poorly mapped, and the influence of ecological changes such as climate‐driven shifts in tick populations on LGTV prevalence and transmission dynamics is unknown. Although generally considered less pathogenic than tick‐borne encephalitis virus, experimental and historical data show that LGTV can cause neurological disease in humans and animals, raising questions about its true risk profile.

The development of LGTV‐based live‐attenuated and chimaeric vaccines has illustrated the difficulty of balancing safety and immunogenicity. While LGTV strains such as E5 and its derivatives showed some promising results in vaccine trials and oncolytic therapy, LGTV can cause disease in vulnerable individuals such as leukemia patients. Still, LGTV remains a valuable experimental model for TBE research.

## Author Contributions

**Zinaida Klestova:** collection of scientific data, analysis, conceptualisation of the review, drafting of the manuscript (original draft writing, review and editing), project administration. **Daniel Sauter:** conceptualisation of the review, visualisation, critical review of the manuscript, optimization, writing, review and editing. **Michael Schindler:** conceptualisation of the review, review and drafting of the manuscript, editing. **Thomas Iftner:** analysis of results, consultation in improving the text and data presentation, critically reviewed the manuscript, editing.

## Funding Statement

Z.K. was supported by a fellowship of the German Academic Exchange Service (DAAD).

## Ethics Statement

The authors have nothing to report.

## Consent

The authors have nothing to report.

## Conflicts of Interest

The authors declare no conflicts of interest.

## Permission to Reproduce Material From Other Sources

The authors have nothing to report.

## Data Availability

Data sharing not applicable to this article as no datasets were generated or analysed during the current study.
